# Comparison of computed tomography–guided core needle biopsy of pulmonary nodules performed with and without the coaxial technique

**DOI:** 10.20452/wiitm.2024.17917

**Published:** 2024-12-02

**Authors:** Qun-Qun Zhu, Li Zhang, Fengfei Xia, Yi‑Bing Shi, Lei Zhu, Xian‑Xian Liang

**Affiliations:** Department of Medical Imaging, The Second Affiliated Hospital of Xuzhou Medical University, Xuzhou, China; Department of Interventional Medicine, Binzhou People’s Hospital, Binzhou, China; Department of Radiology, Xuzhou Central Hospital, Xuzhou, China

**Keywords:** biopsy, coaxial, computed tomography, pulmonary nodule

## Abstract

**INTRODUCTION:**

Computed tomography (CT)-guided core needle biopsy (CNB) is a common method for diagnosing pulmonary nodules (PNs). It is often performed using the coaxial technique (CAT) to streamline the process.

**AIM:**

This study aimed to compare the safety and diagnostic performance of CT-guided CNB with and without CAT for diagnosing PNs.

**MATERIALS AND METHODS:**

This retrospective analysis included patients undergoing CT-guided CNB for a diagnosis of PNs between January 2017 and December 2019. The study population was divided according to the use of CAT for the biopsy. Procedure-related data, diagnostic accuracy and yield, and complication rates were compared between the 2 groups.

**RESULTS:**

During the study period, a total of 111 and 108 patients underwent CT-guided CNB with and without CAT, respectively. As compared with the non-CAT group, the CAT group showed a lower mean needle pathway number (*P* <⁠0.001), a higher mean sample number (*P* <⁠0.001), and shorter procedural duration (*P *<⁠0.001). Diagnostic accuracy was similar between the 2 groups (98.3% vs 96.3%, respectively, for CAT vs non-CAT; *P* = 0.6), though the CAT group demonstrated a higher diagnostic yield than the non-CAT group (81.4% vs 68.5%; *P* = 0.03). Pneumothorax and pulmonary hemorrhage rates did not differ between the 2 groups (*P* = 0.09 and *P* = 0.16, respectively).

**CONCLUSIONS:**

CT-guided CNB with CAT demonstrated greater procedural efficiency, with fewer needle pathways, shorter operative duration, and improved diagnostic yield, as compared with procedures performed without CAT.

## INTRODUCTION 

Computed tomography (CT) is the primary imaging modality used for early‑stage lung cancer screening. Its widespread application resulted in an approximate 20% reduction in lung cancer mortality rates.^1^ On CT scans, early‑stage lung tumors typically manifest as pulmonary nodules (PNs).^2^^;^^3^^;^^4^ CT‑guided core needle biopsy (CNB) is a minimally invasive procedure with high diagnostic accuracy, making it particularly valuable for diagnosing PNs in inoperable cases.^5^^;^^6^^;^^7^

Optimizing sample quality and quantity during biopsy is essential for improving diagnostic yield and accuracy.^8^ However, CNB procedures carry a risk of complications, such as pneumothorax and pulmonary hemorrhage, which are often associated with the need for repeated punctures.^9^ The coaxial technique (CAT) is commonly employed in CT‑guided CNB to reduce the risks associated with multiple punctures.^2^^;^^10^ This method enables repeated sampling without additional punctures. Despite its potential benefits, the impact of CAT on diagnostic accuracy and yield of CT‑guided CNB remains unclear due to limited data. To date, no study has directly compared the outcomes of CT‑guided CNB procedures performed with and without CAT.

## AIM

The present study aimed to evaluate the impact of CAT on the safety and diagnostic efficacy of CT‑guided CNB procedures performed for PN diagnosis.

## MATERIALS AND METHODS

Study design Consecutive patients with PNs undergoing CT‑guided CNB in our hospital between January 2017 and December 2019 were included in the study. We began to use the CAT in our hospital as of May 2018. Consequently, the individuals treated between January 2017 and April 2018 underwent CT‑guided CNB without CAT, and those admitted between May 2018 and December 2019 under went CT‑guided CNB with CAT.

The inclusion criteria comprised: 1) presence of PNs requiring CT‑guided CNB diagnosis, 2) PN size of 8 to 30 mm, and 3) an intermediate to high malignancy risk based on radiological and clinical findings.^11^ The patients with 1) a history of prior CT‑guided CNB, 2) PNs stable in size for at least 2 years, 3) PNs whose dimensions reduced during follow‑up, or 4) insufficient final diagnostic information were excluded from the study.

### Computed tomography–guided core needle biopsy without the coaxial technique

All CNB procedures were performed by an interventional radiolo gist with 10 years of experience (X‑XL). A 16‑row CT scanner (Philips Healthcare, Cleveland, Ohio, United States) was used, with respective tube volt age, tube current, and scanning thickness values of 120 kV, 150 mA, and 2 mm. The needle path ways and positioning were established based on PN location. The needle pathways were determined in such a way so as to minimize the distance between the target nodules and the pleura, while avoiding large vessels, bronchi, fissures, and emphysema. Measures were also implemented to prevent the occurrence of necrotic regions within the target nodules.

Location of the target PNs was determined based on an initial CT scan, with marks on the skin being used for puncture site localization. After the administration of local anesthesia, an 18‑gauge core needle (Wego, Weihai, China) was used to puncture the lung parenchyma. CT was performed again to confirm the correct needle position. The sample was collected from the target nodule after confirming contact between the needle tip and the PN.

For CT‑guided CNB without CAT, a single sample of 5 to 10 cm in length was considered effective. After collection, the samples were transferred into 10% formaldehyde for pathological examination.

### Computed tomography–guided core needle biopsy with the coaxial technique 

The basic protocol of the CNB procedures with CAT was similar to that followed during the non‑CAT biopsies. A 17‑gauge outer needle (DuoSmart, Modena, Italy) was used for the CAT procedure. After establishing contact between the tip of the 17‑gauge needle and the target nodule, an 18‑gauge core needle was inserted through the outer needle to allow sample collection. A total of 3 to 4 samples were collected for each PN using this strategy. After collection, the samples were transferred into 10% formaldehyde for pathological assessment.

### Assessment 

The CT‑guided CNB procedure was considered successful if it allowed for obtaining a pathological diagnosis based on the collected samples, while technical failure was defined as the presence of alveolar or necrotic tissue in the collected sample.^12^

CNB‑based pathological diagnoses of target nodules were classified as 1) malignant, 2) suspected malignant, 3) specific benign lesion, or 4) nonspecific benign lesion. Suspected malignancy diagnoses were based on the presence of atypical cells within the sample with possible or indicated malignancy.^2^^;^^12^ Specific benign lesions comprised tuberculosis, mycotic infections, and benign tumors,^2^^;^^12^ while nonspecific benign lesions were those with benign characteristics on pathological examination for which insufficient information was available to establish a formal diagnosis.^2^^;^^12^

Each PN received a final diagnosis of malignant or benign. Surgical tumor resection results or the acceptance of a CNB‑based malignancy diagnosis were used to determine the final diagnosis of malignancy. A final diagnosis of benignity was made based on either the results of surgical tumor resection, the acceptance of a CNB‑based diagnosis of benignity, a reduction in PN size by at least 20% without any anticancer treatment, or stable lesion size for a minimum of 24 months without any anticancer treatment.^2^^;^^12^

True‑positive and true‑negative results comprised lesions with a CNB‑based diagnosis of malignancy / suspected malignancy or a CNB‑based diagnosis of specific / nonspecific benignity that were respectively confirmed to be malignant and benign on final diagnosis. Diagnostic yield was calculated as follows: (the number of cases with CNB‑based malignancy + the number of cases with CNB‑based specific benignity) / number of all cases. Diagnostic accuracy was calculated as follows: (the number of true‑positive cases + the number of true‑negative cases) / number of all cases.

CT was used to evaluate the patients for complications related to the CNB procedure. The 2 most frequently observed complications were pneumothorax and pulmonary hemorrhage. Pulmonary hemorrhage was defined as evidence of new consolidating / ground‑glass opacity surrounding the needle tract, with high‑grade hemorrhage was defined as needle tract hemorrhage greater than 2 cm in width.^2^

**FIGURE 1:**
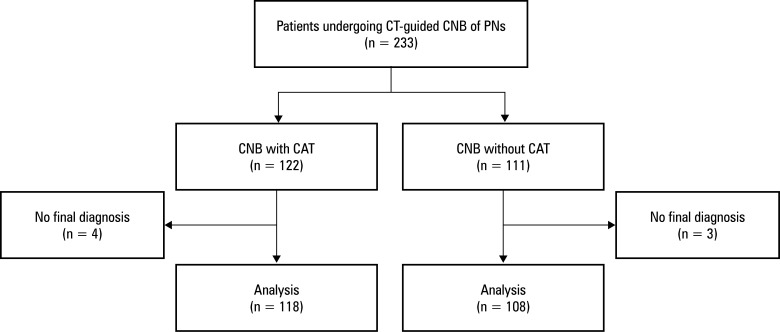
Abbreviations: CAT, coaxial technique; CNB, core needle biopsy; CT, computed tomography; PN, pulmonary nodule

### Statistical analysis 

Normally distributed continuous data were presented as means with SD and compared with the *t* tests, while non‑normally distributed continuous data were presented as median with interquartile range and compared using the Mann–Whitney test. Normality of the data distribution was verified with the *F *test. Categorical data were presented as numbers (percentages) and compared with the χ^2^ test or Fisher exact test. Risk factors related to procedural diagnostic performance and complication rates were detected through logistic regression analyses. Factors identified as significant in univariable analyses (*P* <0.1) were subsequently included in the multivariable models. The threshold for statistical significance was set at *P* below 0.05. All statistical calculations were performed using the SPSS 16.0 package (SPSS Inc., Chicago, Illinois, United States).

### Ethics

The study protocol was approved by the Ethics Committee of Xuzhou Central Hospital (XZXY‑LK‑20230912‑0151), and a requirement for written informed consent was waived due to the retrospective study design.

## RESULTS

### Patients

A total of 233 patients with PNs were enrolled in this study. They were divided into the CAT group (n = 122) and the non‑CAT group (n = 111), according to the CNB technique. Seven patients were excluded due to an absence of a definitive diagnosis, resulting in 111 and 108 patients in the CAT and non‑CAT groups, respectively FIGURE 1. All participants underwent CNB of a single target PN, with a technical success rate of 100% across the entire cohort. Baseline characteristics of the study population are shown inTABLE 1.

### Computed tomography–guided core needle biopsy procedures 

Comparative CNB data are presented in TABLE 2 The CAT group required fewer needle pathways than the non‑CAT group (mean [SD], 1.3 [0.7] vs 1.9 [0.6]; *P* <0.001), and the mean (SD) number of samples was also higher in the CAT group (3.3 [0.5] vs 1.5 [0.4]; *P* <0.001). The mean (SD) procedure time was shorter in the CAT group than in the non‑CAT group (8.9 [2.5] min vs 15.7 [4.4] min; *P* <0.001).

### Diagnostic performance 

No significant differences were observed between the 2 groups in the distribution of CNB‑based or final diagnoses TABLE 3 . Among the 111 patients in the CAT group, 91 CNB‑based malignancies and 5 specific benign lesions were confirmed as final diagnoses. Of the 22 nonspecific benign lesions, 20 were confirmed as benign on follow‑up CT (n = 14) or after surgical resection (n = 6), while 2 were confirmed as malignant after surgical resection.

In the non‑CAT group, 69 cases of CNB‑based malignancy and 5 cases of specific benignity were accepted as final diagnoses. Surgical resection confirmed the malignancy of 2 CNB‑based suspected malignancies, while of the 32 CNB‑based nonspecific benign lesions, 28 were confirmed to be truly benign based on surgical resection (n = 8) or follow‑up CT (n = 20) findings, whereas 4 cases were found to be malignant on surgical resection.

Diagnostic accuracy was comparable between the CAT and non‑CAT groups (98.3% vs 96.3%; *P* = 0.6); however, the CAT group demonstrated a higher diagnostic yield (81.4% vs 68.5%; *P *= 0.03). Diagnostic failure was not associated with any risk factors identified in the univariate analysis. Molecular testing was needed for 75 patients in the CAT group and 53 patients in the non‑CAT group. The adequacy rates for molecular testing in the CAT group were higher than those observed in the non‑CAT group (98.7% vs 88.7%; *P* = 0.04).

**TABLE 1:** Baseline characteristics of the study population

Parameter		CNB with CAT (n = 118)	CNB without CAT (n = 108)	*P *value
Age, y		63.5 (12.9)	60.4 (11.1)	0.06
Sex	Men	64 (54.2)	54 (50)	0.52
	Women	54 (45.8)	54 (50)	
Smoking history		27 (22.9)	42 (38.9)	0.009
Emphysema		17 (14.4)	26 (24.1)	0.06
BMI, kg/m^2^		22.8 (3.4)	23.1 (3.4)	0.5
Lesion size, mm		18.9 (5.4)	17.8 (4.5)	0.09
Affected lung	Left	44 (37.3)	57 (52.8)	0.02
	Right	74 (62.7)	51 (47.2)	
Affected lung lobe	Upper	59 (50)	45 (41.7)	0.21
	Nonupper	59 (50)	63 (58.3)	

Data are presented as number (percentage) of patients or mean (SD).

Abbreviations: BMI, body mass index; others, see FIGURE 1

**TABLE 2:** Comparison of procedural data between 2 groups

Parameter		CNB with CAT (n = 118)	CNB without CAT (n = 108)	*P *value
Procedural characteristics				
Intrapulmonary needle length, mm, median (IQR)		14 (8–30)	13 (6.3–23)	0.19
Needle-pleura angle, degree		75.3 (16.5)	70.1 (17.7)	0.03
Patient position	Prone	69 (58.5)	80 (74.1)	0.03
	Supine	48 (40.7)	26 (24.1)	
	Decubitus	1 (0.8)	2 (1.8)	
Needle pathways, n		1.3 (0.7)	1.9 (0.6)	<0.001
Samples, n		3.3 (0.5)	1.5 (0.4)	<0.001
Procedure duration, min		8.9 (2.5)	15.7 (4.4)	<0.001
Complications				
Pneumothorax		24 (20.3)	13 (12)	0.09
Pneumothorax requiring chest tube insertion		1 (0.8)	6 (5.6)	0.1
Pulmonary hemorrhage		43 (36.4)	30 (27.8)	0.16
High-grade pulmonary hemorrhage		28 (23.7)	15 (13.9)	0.06

Data are presented as number (percentage) of patients or mean (SD) unless indicated otherwise.

Abbreviations: IQR, interquartile range; others, see FIGURE 1

### Complications

Pneumothorax occurred in 24 patients (20.3%) in the CAT group and 13 patients (12%) in the non‑CAT group (*P *= 0.09). Seven individuals required chest tube insertion (1 in the CAT group and 6 in the non‑CAT group). All other patients were managed conservatively. In univariable and multivariable analyses, the use of CAT was identified as a risk factor for pneumothorax (*P* = 0.04)TABLE 4 .

The rates of pulmonary hemorrhage were 36.4% (n = 43) and 27.8% (n = 30) in the CAT and non‑CAT groups, respectively (*P* = 0.16). High grade hemorrhage occurred in 28 and 15 patients in the CAT and non‑CAT groups, respectively (*P* = 0.06). Conservative treatment was used to manage all pulmonary hemorrhage episodes. In univariable and multivariable analyses TABLE 5 , greater intrapulmonary needle length was found to correlate with an increased risk of high‑grade pulmonary hemorrhage (*P* = 0.001).

## DISCUSSION 

This study compared the safety and diagnostic efficacy of CT‑guided CNB procedures for PN diagnosis, performed with and without CAT. The use of CAT enables repeated sampling from target lesions through an esablished pathway.^2^ In this study, its application was associated with a lower number of needle pathways (mean [SD], 1.3 [0.7] vs 1.9 [0.6]; *P* <0.001) and a greater mean (SD) number of samples collected (3.3 [0.5] vs 1.5 [0.4]; *P* <0.001), as compared with the procedures performed without the CAT. The use of CAT also decreased the CNB duration (mean [SD], 8.9 [2.5] min vs 15.7 [4.4] min, respectively, for CAT vs non‑CAT;* P* <0.001).

Diagnostic yield reflects the ability of CT‑guided CNB to provide definitive diagnoses, while diagnostic accuracy indicates the reliability of these results.^2^ Clinicians can only accept CNB‑based malignancy and specific benignity diagnoses as the final diagnosis for evaluated patients.^13^ Here, CNB procedures performed with the CAT were associated with a significant improvement in diagnostic yield, as compared with procedures performed without this technique (81.4% vs 68.5%; *P* = 0.03). This observation may be partially attributed to the higher prevalence of malignant lesions observed in the non‑CAT group. Moreover, 2 cases of suspected malignancy based on CNB findings were observed in the non‑CAT group, while the CAT group featured no such cases. Despite the limited statistical power of these analyses, this may indicate that the rates of suspected malignant diagnoses based on CNB could potentially be reduced via the use of CAT.

High diagnostic accuracy was achieved in both groups (98.3% vs 96.3%, respectively, for CAT vs non‑CAT; *P* = 0.6), comparable to the rates reported in prior studies (93%–96.1%),^14^^;^^15^^;^^16^ indicating that CAT did not significantly enhance the precision of CT‑guided CNB for diagnosing PNs. The high diagnostic accuracy rates for CNB procedures performed with CAT are attributable to the collection of 3 to 4 samples per 1 PN. Although fewer samples were collected during the non‑CAT procedures, each sample underwent strict quality assessment, ensuring a high level of diagnostic precision.

The use of CAT did not demonstrate any correlation with enhanced diagnostic accuracy of CNB when evaluating PNs. Currently, CNB procedures serve 2 roles: they assist in the pathological diagnosis of target lesions and promote molecular testing efforts.^17^ In this study, CAT implementation was found to be superior for molecular testing due to its ability to obtain additional samples from the target nodules.

**TABLE 3:** Comparison of diagnostic performance between the 2 groups

Parameter	CNB with CAT (n = 118)	CNB without CAT (n = 108)	*P *value
Biopsy-based pathological diagnosis			
Malignancy	91 (77.1)	69 (63.9)	0.09
Suspected malignancy	0	2 (1.9)	
Specific benign lesion	5 (4.3)	5 (4.6)	
Nonspecific benign lesion	22 (18.6)	32 (29.6)	
Final diagnosis			
Malignancy	93 (78.8)	75 (69.4)	0.11
Benignity	25 (21.2)	33 (30.6)	
Diagnostic performance^a^			
Diagnostic yield, %	81.4 (96/118)	68.5 (74/108)	0.03
Diagnostic accuracy, %	98.3 (116/118)	96.3 (104/108)	0.6
Adequacy for molecular test, %	98.7 (74/75)	88.7 (47/53)	0.04

Data are presented as number (percentage) of patients unless indicated otherwise.

a The calculation method is described in detail in the *Materials and Methods* section.

Abbreviations: see FIGURE 1

**TABLE 4:** Predictors of pneumothorax

Variable		Univariable analysis				Multivariable analysis	
		OR	95% CI	*P *value	OR	95% CI	*P* value
Age		1.02	0.999–1.052	0.21	–	–	–
Sex	Men	1 (ref.)	–	–	1 (ref.)	–	–
	Women	0.344	0.158–0.75	0.007	0.595	0.231–1.529	0.28
Smoking history		2.934	1.427–6.036	0.003	2.239	0.833–6.02	0.11
Emphysema		2.446	1.112–5.383	0.03	1.496	0.582–3.842	0.4
BMI		0.953	0.856–1.061	0.38	–	–	–
Lesion size		1.05	0.977–1.127	0.185	–	–	–
Affected lung	Right	1 (ref.)	–	–	–	–	–
	Left	0.817	0.399–1.671	0.58	–	–	–
Affected lung lobe	Nonupper	1 (ref.)	–	–	–	–	–
	Upper	0.583	0.28–1.214	0.15	–	–	–
Intrapulmonary needle length		1.006	0.983–1.03	0.593	–	–	–
Needle-pleura angle		1	0.98–1.021	0.986	–	–	–
Patient position	Prone	1 (ref.)	–	–	–	–	–
	Nonprone	1.659	0.829–3.323	0.15	–	–	–
Number of needle pathways		0.9	0.542–1.496	0.69	–	–	–
Procedure duration		1.032	0.963–1.106	0.37	–	–	–
Use of CAT	Yes	1 (ref.)	–	–	1 (ref.)	–	–
	No	0.536	0.258–1.115	0.095	0.429	0.195–0.945	0.036

Abbreviations: OR, odds ratio; ref., reference; others, see FIGURE 1 and TABLE 1

Patient safety was not compromised by CAT use, though it was identified as a risk factor for pneumothorax, likely due to the larger diameter of the outer needle. Nevertheless, the CAT group showed a lower rate of pneumothorax cases requiring chest tube insertion than the non‑CAT group (0.8% vs 5.6%; *P* = 0.1), likey due to the reduced number of needle pathways. Greater intrapulmonary needle lengths correlated with an increased risk of high‑grade pulmonary hemorrhage, consistent with prior findings.^2^^;^^15^

Bronchoscopy‑guided lung biopsy or PN localization has also been widely used as a diagnostic method.^18^^;^^19^ As compared with the CT‑guided approach, the bronchoscopic approach was associated with better safety.^18^^;^^19^ However, bronchoscopy‑guided lung biopsy exhibited significantly lower diagnostic yield and accuracy than CT‑guided biopsy.^19^

**TABLE 5:** Predictors of high-grade pulmonary hemorrhage

Variable		Univariable analysis				Multivariable analysis	
		OR	95% CI	*P* value	OR	95% CI	*P* value
Age		1.015	0.987–1.045	0.29	–	–	–
Sex	Men	1 (ref.)	–	–	–	–	–
	Women	0.794	0.409–1.542	0.5	–	–	–
Smoking history		0.994	0.459–1.939	0.87	–	–	–
Emphysema		1.119	0.492–2.546	0.79	–	–	–
BMI		1.029	0.935–1.132	0.56	–	–	–
Lesion size		0.987	0.923–1.053	0.68	–	–	–
Affected lung	Right	1 (ref.)	–	–	–	–	–
	Left	0.927	0.477–1.801	0.82	–	–	–
Affected lung lobe	Nonupper	1 (ref.)	–	–	–	–	–
	Upper	1.716	0.883–3.335	0.11	–	–	–
Intrapulmonary needle length		1.038	1.016–1.06	0.001	1.037	1.014–1.061	0.001
Needle-pleura angle		1.022	0.999–1.045	0.061	1.021	0.998–1.045	0.08
Patient position	Prone	1 (ref.)	–	–	–	–	–
	Nonprone	1.324	0.885–2.559	0.4	–	–	–
Number of needle pathways		1.137	0.733–1.765	0.57	–	–	–
Procedure duration		0.985	0.999–1.036	0.33	–	–	–
Use of CAT	Yes	1	–	–	1	–	–
	No	0.495	0.249–0.985	0.045	0.254	0.315–1.357	0.25

Abbreviations: see FIGURE 1,TABLE 1, and TABLE 4

The limitations of this study include a retrospective design, which is inherently associated with a high risk of selection bias. Moreover, baseline differences between the groups with respect to the affected lung side and smoking history were not balanced between 2 groups. Some patients were excluded due to an absence of any final diagnosis, which may have influenced the calculated diagnostic accuracy.

## CONCLUSIONS

CT‑guided CNB procedures using CAT were more efficient, with fewer needle pathways, shorter operative times, and improved diagnostic yield, as compared with those performed without CAT. These findings support the use of CAT as a valuable tool for optimizing CT‑guided CNB procedures.

## IBUTION STATEMENT

X-XL conceived the concept of the study. X-XL and LZhang contributed to the design of the research. All authors were involved in data collection. QQZ and LZhu analyzed the data. All authors edited and approved the final version of the manuscript.
